# Supplementary material for: The Impact of COVID-19 Pandemic on Inequity in Routine Childhood Vaccination Coverage: A Systematic Review
Source: Vaccines (Basel). 2022 Jun 24;10(7):1013. doi: 10.3390/vaccines10071013 (PMC9321080; doi:10.3390/vaccines10071013)
Supplement: Supplementary file 1 [file vaccines-10-01013-s001.zip › supplementary material vaccines spencer et al.pdf]

SUPPLEMENTARY MATERIAL

File S1: Protocol registered with PROSPERO (CRD 42021257431)

File S2: Search strategy and numbers for each electronic database

Initial search :01 Jan 2020 – 30 April 2021)

Date of search: 30 April 2021

	Nos. references	References after de-duplication
Medline ALL	122	113
Embase	125	93
Web of Science	45	11
Cochrane CENTRAL	1	1
Cochrane CDSR	1	1
Sociological Abstracts	0	0
ASSIA	5	5
MedRxriv	667	664
WHO	8	0
**TOTAL**	**974**	**888**

Date of updated search: 20 January 2022

	Nos. references	References after de-duplication
Medline ALL	248	246
Embase	329	247
Web of Science	101	27
Cochrane CENTRAL	4	3
Cochrane CDSR	0	0
Sociological Abstracts	7	6
ASSIA	40	36
WHO	12	0
TOTAL	741	565

Initial and updated search used the identical search strategies for all databases except MedRxiv (indexed in PubMed from February 2020):

Database: Ovid MEDLINE(R) ALL <01 Jan 2020 to 30 April, 2021 & 1 May to 20 January 2022 >
Search Strategy:
----
1 exp Coronavirus/ (69808)
2 exp Coronavirus Infections/ (84886)
3 (coronavirus* or corona virus* or OC43 or NL63 or 229E or HKU1 or HCoV* or ncov* or covid* or sars-cov* or sarscov* or Sars-coronavirus* or Severe Acute Respiratory Syndrome Coronavirus* or "Kawasaki like paediatric inflammatory multisystem syndrome" or "Kawasaki like pediatric inflammatory multisystem syndrome" or "PIMS-TS" or "Kawa-COVID-19" or "MIS-C" or "multisystem inflammatory syndrome in children" or pediatric multisystem inflammatory disease).mp. (145733)
4 (or/1-3) and ((20191* or 202*).dp. or 20190101:20301231.(ep).) [this set is the sensitive/broad part of the search] (132747)
5 4 not (SARS or SARS-CoV or MERS or MERS-CoV or Middle East respiratory syndrome or camel* or dromedar* or equine or coronary or coronal or covidence* or covidien or influenza virus or HIV or bovine or calves or TGEV or feline or porcine or BCoV or PED or PEDV or PDCoV or FIPV or FCoV or SADS-CoV or canine or CCov or zoonotic or avian influenza or H1N1 or H5N1 or H5N6 or IBV or murine corona*).mp. [line 5 removes SARS, MERS and veterinary noise from the sensitive/broad search results] (49778)
6 ((pneumonia or covid* or coronavirus* or corona virus* or ncov* or 2019-ncov or sars*).mp. or exp pneumonia/) and Wuhan.mp. [Early articles about the outbreak] (5028)
7 (2019-ncov or ncov19 or ncov-19 or 2019-novel CoV or sars-cov2 or sars-cov-2 or sarscov2 or sarscov-2 or SARS-2-nCoV or SARS-2-Cov or SARS-COV-19 or Sars-coronavirus2 or Sars-coronavirus-2 or SARS 2 coronavirus* or Severe Acute Respiratory Syndrome-CoV-2 or SARS-like coronavirus* or coronavirus-19 or covid19 or covid-19 or covid 2019 or ((novel or new or nouveau) adj2 (CoV or nCoV or covid or coronavirus* or corona virus or Pandemi*2)) or ((covid or covid19 or covid-19 or SARS-CoV-2) and pandemic*2) or (coronavirus* and pneumonia)).mp. [specific to Covid-19, Covid pneumonia, Covid pandemic] (130870)
8 or/1-7 (151701)
9 exp Vaccines/ or vaccin*.mp. or exp Vaccination/ (405200)
10 exp mass vaccination/ (3194)
11 exp viral vaccines/ or viral vaccin*.mp. (116800)
12 exp vaccination coverage/ (1527)
13 exp Immunization Programs/ or exp Immunization/ or immunisation.mp. (193014)
14 immuni*.mp. (470987)
15 9 or 10 or 11 or 12 or 13 or 14 (747928)
16 inequality.mp. or exp Socioeconomic Factors/ (478738)
17 inequalit*.mp. (38595)
18 exp Poverty/ or poverty.mp. (63017)
19 healthcare disparities.mp. or exp Healthcare Disparities/ (19556)
20 exp Health Services Accessibility/ (115944)
21 exp Income/ or income.mp. (184412)
22 exp Social Class/ or social status.mp. or social class.mp. (52640)
23 exp Educational Status/ or educat*.mp. [mp=title, abstract, original title, name of substance word, subject heading word, floating sub-heading word, keyword heading word, organism

supplementary concept word, protocol supplementary concept word, rare disease supplementary concept word, unique identifier, synonyms] (1079487)
24 exp Health Status Disparities/ or exp Health Status/ (360714)
25 16 or 17 or 18 or 19 or 20 or 21 or 22 or 23 or 24 (1871410)
26 exp Child/ or child*.mp. (2521414)
27 infant*.mp. or exp Infant/ (1299261)
28 26 or 27 (3066826)
29 8 and 15 and 25 and 28 (135)
30 limit 29 to yr="2020 -Current" (122)

Database: Embase <2020 Week 1 to 2021 Week 16 & 2021 Week 17 to 2022 Week3>
Search Strategy:
----
1 exp Coronavirinae/ (46704)
2 exp Coronavirus infection/ (125467)
3 ("coronavirus disease 2019" or "severe acute respiratory syndrome coronavirus 2").sh,dj. (109570)
4 ((corona* or corono*) adj1 (virus* or viral* or virinae*)).ti,ab,kw. (2595)
5 (coronavirus* or coronovirus* or coronavirinae* or CoV).ti,ab,kw. (80523)
6 ("2019-nCoV*" or 2019nCoV* or "19-nCoV*" or 19nCoV* or nCoV2019* or "nCoV-2019*" or nCoV19* or "nCoV-19*" or "COVID-19*" or COVID19* or "COVID-2019*" or COVID2019* or "HCoV-19*" or HCoV19* or "HCoV-2019*" or HCoV2019* or "2019 novel*" or Ncov* or "n-cov" or "SARS-CoV-2*" or "SARSCoV-2*" or "SARSCoV2*" or "SARS-CoV2*" or SARSCov19* or "SARS-Cov19*" or "SARSCov-19*" or "SARS-Cov-19*" or SARSCov2019* or "SARS-Cov2019*" or "SARSCov-2019*" or "SARS-Cov-2019*" or SARS2* or "SARS-2*" or SARScoronavirus2* or "SARS-coronavirus-2*" or "SARScoronavirus 2*" or "SARS coronavirus2*" or SARScoronovirus2* or "SARS-coronovirus-2*" or "SARScoronovirus 2*" or "SARS coronovirus2*" or covid).ti,ab,kw. (118765)
7 (respiratory* adj2 (symptom* or disease* or illness* or condition*) adj5 (Wuhan* or Hubei* or China* or Chinese* or Huanan*)).ti,ab,kw. (403)
8 (("seafood market*" or "food market*") adj10 (Wuhan* or Hubei* or China* or Chinese* or Huanan*)).ti,ab,kw. (106)
9 (pneumonia* adj3 (Wuhan* or Hubei* or China* or Chinese* or Huanan*)).ti,ab,kw. (652)
10 ((outbreak* or wildlife* or pandemic* or epidemic*) adj1 (Wuhan* or Hubei* or China* or Chinese* or Huanan*)).ti,ab,kw. (174)
11 "severe acute respiratory syndrome*".ti,ab,kw. (19474)
12 or/1-11 (158644)
13 limit 12 to yr="2019 -Current" (135192)
14 limit 13 to medline (29810)
15 13 not 14 (105382)
16 exp vaccination/ or vaccin*.mp. or exp vaccine/ (487379)
17 mass vaccination.mp. or exp mass immunization/ (4974)
18 exp virus vaccine/ or viral vaccine*.mp. (158174)
19 exp vaccination coverage/ (2769)
20 exp immunization/ (284427)
21 immuni*.mp. (599955)
22 16 or 17 or 18 or 19 or 20 or 21 (916665)

23 inequalit*.mp. or exp inequality/ or exp socioeconomics/ (428529)
24 exp poverty/ or poverty.mp. (60661)
25 exp Healthcare Disparities/ or healthcare disparit*.mp. (18297)
26 health services accessibility.mp. or exp health care access/ (70230)
27 exp income group/ or exp household income/ or exp family income/ or income.mp. or exp income/ (235109)
28 exp social class/ or social class.mp. (34744)
29 exp educational status/ (82957)
30 exp health status/ (256430)
31 socioeconomic*.mp. or exp social status/ (359344)
32 23 or 24 or 25 or 26 or 27 or 28 or 29 or 30 or 31 (956674)
33 exp child/ or child*.mp. (3181748)
34 exp infant/ or infant*.mp. (1150614)
35 33 or 34 (3275646)
36 15 and 22 and 32 and 35 (128)
37 limit 36 to yr="2020 -Current" (125)

Web of Science

# 5	45	#4 AND #3 AND #2 AND #1<br>*Indexes=SCI-EXPANDED, SSCI, CPCI-S, CPCI-SSH Timespan=2020-2021*
# 4	145,119	**TOPIC:** (child* or infant*)<br>*Indexes=SCI-EXPANDED, SSCI, CPCI-S, CPCI-SSH Timespan=2020-2021*
# 3	74,234	**TOPIC:**<br>(inequalit* or socioeconomic* or poverty or "healthcare disparit*" or "health service access*" or income or "social class*" or "social status" or "educational status" or "health status")<br>*Indexes=SCI-EXPANDED, SSCI, CPCI-S, CPCI-SSH Timespan=2020-2021*
# 2	51,856	**TOPIC:** (vaccin* or immuni*)<br>*Indexes=SCI-EXPANDED, SSCI, CPCI-S, CPCI-SSH Timespan=2020-2021*
# 1	100,169	TS=(coronavirus* or coronaviridae or covid* or 2019-ncov or ncov19 or ncov-19 or 2019-novel CoV or sars-cov2 or sars-cov-2 or sarscov2 or sarscov-2 or SARS-2-nCoV or SARS-2-Cov or SARS-COV-19 or Sars-coronavirus2 or Sars-coronavirus-2 or SARS 2 coronavirus* or Severe Acute Respiratory Syndrome-CoV-2 or SARS-like coronavirus* or coronavirus-19 or covid19 or covid-19 or covid 2019) or TS=((novel or new or nouveau) AND (CoV or nCoV or covid or coronavirus* or corona virus or Pandemi*)) or TS= ((covid or covid19 or covid-19 or SARS-CoV-2) and pandemic*) or TS=(coronavirus* and pneumonia)<br>*Indexes=SCI-EXPANDED, SSCI, CPCI-S, CPCI-SSH Timespan=2020-2021*

The Cochrane Library (Numbers refer to 30/04/2021 run)
Date Run: 30/04/2021 & 20/01/2022
ID Search Hits
#1 MeSH descriptor: [Coronavirus] explode all trees 264
#2 MeSH descriptor: [Coronavirus Infections] explode all trees 828
#3 MeSH descriptor: [Coronaviridae] explode all trees 266
#4 (Covid* or covid-19 or "covid19" or SARS-COV-2 or coronavirus* or "corona virus*" or sars-cov* OR sarscov* or sars-coronavirus*):ti,ab,kw 6210
#5 #1 or #2 or #3 or #4 6231
#6 MeSH descriptor: [Vaccines] explode all trees 13126
#7 MeSH descriptor: [Vaccines] explode all trees 13126
#8 MeSH descriptor: [Mass Vaccination] explode all trees 37
#9 MeSH descriptor: [Viral Vaccines] explode all trees 5288
#10 MeSH descriptor: [Vaccination Coverage] explode all trees 26

#11 MeSH descriptor: [Immunity] explode all trees 4024
#12 MeSH descriptor: [Immunization Programs] explode all trees 219
#13 (vaccin* or immuni*):ti,ab,kw 33243
#14 #6 or #7 or #8 or #9 or #10 or #11 or #12 or #13 34334
#15 #5 and #14 640
#16 MeSH descriptor: [Socioeconomic Factors] explode all trees 9960
#17 MeSH descriptor: [Poverty] explode all trees 1766
#18 MeSH descriptor: [Healthcare Disparities] explode all trees 187
#19 MeSH descriptor: [Health Services Accessibility] explode all trees 981
#20 MeSH descriptor: [Income] explode all trees 989
#21 MeSH descriptor: [Social Class] explode all trees 651
#22 MeSH descriptor: [Educational Status] explode all trees 1476
#23 MeSH descriptor: [Health Status Disparities] explode all trees 167
#24 MeSH descriptor: [Social Determinants of Health] explode all trees 22
#25 (inequalit* or poverty or social status):ti,ab,kw 13342
#26 MeSH descriptor: [Health Status] explode all trees 30702
#27 #16 or #17 or #18 or #19 or #20 or #21 or #22 or #23 or #24 or #25 or #26 49351
#28 #15 and #27 16
#29 (child* or infant*):ti,ab,kw 192664
#30 #28 and #29 2

Sociological Abstracts.

noft(Covid* OR covid-19 OR "covid19" OR SARS-COV-2 OR coronavirus* OR "corona virus*" OR sars-cov* OR sarscov* OR sars-coronavirus*) AND (MAINSUBJECT.EXACT.EXPLODE("Vaccination") OR noft(vaccin* OR immuni*)) AND ((MAINSUBJECT.EXACT.EXPLODE("Income Inequality") OR MAINSUBJECT.EXACT.EXPLODE("Inequality") OR MAINSUBJECT.EXACT.EXPLODE("Social Inequality") OR MAINSUBJECT.EXACT.EXPLODE("Educational Inequality")) OR (MAINSUBJECT.EXACT.EXPLODE("Socioeconomic Factors") OR MAINSUBJECT.EXACT.EXPLODE("Socioeconomic Status")) OR MAINSUBJECT.EXACT.EXPLODE("Poverty") OR (MAINSUBJECT.EXACT.EXPLODE("Income Inequality") OR MAINSUBJECT.EXACT.EXPLODE("Income")) OR MAINSUBJECT.EXACT.EXPLODE("Social Class") OR noft(inequalit* OR poverty OR income OR "social class" OR "educational status" OR "healthcare disparit*" OR "social status" OR socioeconomic OR "health services access*")) AND noft(infant* or child|*)

ASSIA

noft(Covid* OR covid-19 OR "covid19" OR SARS-COV-2 OR coronavirus* OR "corona virus*" OR sars-cov* OR sarscov* OR sars-coronavirus*) AND ((MAINSUBJECT.EXACT.EXPLODE("Vaccines") OR MAINSUBJECT.EXACT.EXPLODE("Combined vaccines")) OR noft(vaccin* OR immuni*) OR MAINSUBJECT.EXACT.EXPLODE("Immunization")) AND ((MAINSUBJECT.EXACT.EXPLODE("Income inequalities") OR MAINSUBJECT.EXACT.EXPLODE("Inequalities") OR MAINSUBJECT.EXACT.EXPLODE("Health inequalities") OR MAINSUBJECT.EXACT.EXPLODE("Economic inequalities") OR MAINSUBJECT.EXACT.EXPLODE("Social inequalities") OR MAINSUBJECT.EXACT.EXPLODE("Racial inequalities")) OR MAINSUBJECT.EXACT.EXPLODE("Poverty") OR MAINSUBJECT.EXACT.EXPLODE("Social class") OR MAINSUBJECT.EXACT.EXPLODE("Health status")

OR noft(inequalit* OR poverty OR "social class" OR "social status" OR income OR socioeconomic OR "educational status" OR "health status" OR "health services access*" OR "health disparit*")) AND noft(infant* or child*)

WHO Global Research on Corona Virus (COVID-19) accessed 1 May 2021 and 20 January 2022 at (https://www.who.int/emergencies/diseases/novel-coronavirus-2019/global-research-on-novel-coronavirus-2019-ncov using key words: “routine childhood vaccination coverage”.

Characteristics of publication					Population and sampling			Routine childhood vaccines studied	Inequity	Analysis	Results
**No.**	**Country/ Countries studied**	**Citation**	**Research objective**	**Type of study**	**Population & months of COVID pandemic studied**	**Sampling method**	**Sample size**	**Routine childhood vaccines studied**	**Socioeconomic status, poverty/wealth, indigenous group, ethnic group measured at individual &/or regional level)**	**Methods of analysis of inequity and measures of effect used**	**Population-level reductions in routine vaccination coverage by individual vaccine Reductions by inequity measures with effect size estimates where reported**

Table S1: Data extraction spreadsheet headings

Section and Topic	Item #	Checklist item	Location where item is reported [lines]
**TITLE**			
Title	1	Identify the report as a systematic review.	1-2
**ABSTRACT**			
Abstract	2	See the PRISMA 2020 for Abstracts checklist.	28-48
**INTRODUCTION**			
Rationale	3	Describe the rationale for the review in the context of existing knowledge.	52-66
Objectives	4	Provide an explicit statement of the objective(s) or question(s) the review addresses.	67-78
**METHODS**			
Eligibility criteria	5	Specify the inclusion and exclusion criteria for the review and how studies were grouped for the syntheses.	100-106<br>See Panel
Information sources	6	Specify all databases, registers, websites, organisations, reference lists and other sources searched or consulted to identify studies. Specify the date when each source was last searched or consulted.	87-92
Search strategy	7	Present the full search strategies for all databases, registers and websites, including any filters and limits used.	93-99
Selection process	8	Specify the methods used to decide whether a study met the inclusion criteria of the review, including how many reviewers screened each record and each report retrieved, whether they worked independently, and if applicable, details of automation tools used in the process.	108-112
Data collection process	9	Specify the methods used to collect data from reports, including how many reviewers collected data from each report, whether they worked independently, any processes for obtaining or confirming data from study investigators, and if applicable, details of automation tools used in the process.	108-112
Data items	10a	List and define all outcomes for which data were sought. Specify whether all results that were compatible with each outcome domain in each study were sought (e.g. for all measures, time points, analyses), and if not, the methods used to decide which results to collect.	125-130<br>Panel
	10b	List and define all other variables for which data were sought (e.g. participant and intervention characteristics, funding sources). Describe any assumptions made about any missing or unclear information.	Table 1
Study risk of bias assessment	11	Specify the methods used to assess risk of bias in the included studies, including details of the tool(s) used, how many reviewers assessed each study and whether they worked independently, and if applicable, details of automation tools used in the process.	136-139
Effect measures	12	Specify for each outcome the effect measure(s) (e.g. risk ratio, mean difference) used in the synthesis or presentation of results.	
Synthesis methods	13a	Describe the processes used to decide which studies were eligible for each synthesis (e.g. tabulating the study intervention characteristics and comparing against the planned groups for each synthesis (item #5)).	Table 2
	13b	Describe any methods required to prepare the data for presentation or synthesis, such as handling of missing summary statistics, or data conversions.	N/A

Section and Topic	Item #	Checklist item	Location where item is reported [lines]
	13c	Describe any methods used to tabulate or visually display results of individual studies and syntheses.	Tables 1&2
	13d	Describe any methods used to synthesize results and provide a rationale for the choice(s). If meta-analysis was performed, describe the model(s), method(s) to identify the presence and extent of statistical heterogeneity, and software package(s) used.	118-120
	13e	Describe any methods used to explore possible causes of heterogeneity among study results (e.g. subgroup analysis, meta-regression).	N/A
	13f	Describe any sensitivity analyses conducted to assess robustness of the synthesized results.	N/A
Reporting bias assessment	14	Describe any methods used to assess risk of bias due to missing results in a synthesis (arising from reporting biases).	N/A
Certainty assessment	15	Describe any methods used to assess certainty (or confidence) in the body of evidence for an outcome.	122-4
**RESULTS**			
Study selection	16a	Describe the results of the search and selection process, from the number of records identified in the search to the number of studies included in the review, ideally using a flow diagram.	142-4<br>Figure 1
	16b	Cite studies that might appear to meet the inclusion criteria, but which were excluded, and explain why they were excluded.	144-151
Study characteristics	17	Cite each included study and present its characteristics.	153-178
Risk of bias in studies	18	Present assessments of risk of bias for each included study.	187-190<br>Table 3
Results of individual studies	19	For all outcomes, present, for each study: (a) summary statistics for each group (where appropriate) and (b) an effect estimate and its precision (e.g. confidence/credible interval), ideally using structured tables or plots.	Table 2
Results of syntheses	20a	For each synthesis, briefly summarise the characteristics and risk of bias among contributing studies.	Table 1
	20b	Present results of all statistical syntheses conducted. If meta-analysis was done, present for each the summary estimate and its precision (e.g. confidence/credible interval) and measures of statistical heterogeneity. If comparing groups, describe the direction of the effect.	Narrative synthesis:<br>198-336
	20c	Present results of all investigations of possible causes of heterogeneity among study results.	n/a
	20d	Present results of all sensitivity analyses conducted to assess the robustness of the synthesized results.	n/a
Reporting biases	21	Present assessments of risk of bias due to missing results (arising from reporting biases) for each synthesis assessed.	n/a
Certainty of evidence	22	Present assessments of certainty (or confidence) in the body of evidence for each outcome assessed.	339-407

Section and Topic	Item #	Checklist item	Location where item is reported [lines]
**DISCUSSION**			
Discussion	23a	Provide a general interpretation of the results in the context of other evidence.	418-453
	23b	Discuss any limitations of the evidence included in the review.	418-421
	23c	Discuss any limitations of the review processes used.	454-464
	23d	Discuss implications of the results for practice, policy, and future research.	465-482
**OTHER INFORMATION**			
Registration and protocol	24a	Provide registration information for the review, including register name and registration number, or state that the review was not registered.	83-4
	24b	Indicate where the review protocol can be accessed, or state that a protocol was not prepared.	Available at: *https://www.crd.york.ac.uk/**PROSPERO***
	24c	Describe and explain any amendments to information provided at registration or in the protocol.	
Support	25	Describe sources of financial or non-financial support for the review, and the role of the funders or sponsors in the review.	491
Competing interests	26	Declare any competing interests of review authors.	504
Availability of data, code and other materials	27	Report which of the following are publicly available and where they can be found: template data collection forms; data extracted from included studies; data used for all analyses; analytic code; any other materials used in the review.	Supp table S1<br>Obtain from authors

From: Page MJ, McKenzie JE, Bossuyt PM, Boutron I, Hoffmann TC, Mulrow CD, et al. The PRISMA 2020 statement: an updated guideline for reporting systematic reviews. BMJ 2021;372:n71. doi: 10.1136/bmj.n71

Table S2: PRISMA 2020 Checklist
